# Letters from the Virginia Springs

**Published:** 1840-08-01

**Authors:** 


### MEDICAL EXAMINER.

DEVOTED TO MEDICINE, SURGERY, AND THE COLLATERAL SCIENCES.

No. 31.] PHILADELPHIA, SATURDAY, AUGUST 1, 1840. [Vol. III.

### LETTERS FROM THE VIRGINIA SPRINGS.—No. I.

For some years past, a visit to the group of mineral springs scattered through the middle of the mountainous part of Virginia, is often made by invalids for the restoration of a shattered constitution, and by many others who seek relief from the confinement and heat of cities by a visit to a healthy mountainous region, in which the mineral springs offer many other attractions besides their medicinal qualities. With the latter class of visiters a physician has no concern; as their object is relaxation and pleasure, it is of little moment whether they drink the sulphur waters in Virginia, or the saline springs at Saratoga; but, with the invalids the case is widely different, and a neglect of some obvious precautions often neutralizes the good effects of a medicinal spring, and may render the waters, or the waters combined with the journey, positively of injury,—and we often find that patients who have sought for health have returned after a fatiguing and expensive journey in a worse condition than at their departure from home. In fact, a mineral water is a powerful remedy; and if we combine with it a laborious journey, its influence upon an invalid is singularly augmented; whether this be exerted for good or evil, is the province of a physician to determine. It is much to be regretted that agents of this power should be prescribed with so little reflection, to relieve the medical attendant of a troublesome case, or that they should be resorted to by the patient himself without competent advice, and that the means which might have led to health should become the seeds of disease, or should cherish it when already implanted in the system.

In making the journey to the mountain springs of Virginia, every one complains of the fatigue which is caused by it; this is, of course, much greater in the ordinary stage coaches than in private carriages; still, there is no way of avoiding it, and the invalid must regard it as part of the treatment,—whether this be for good or evil, the nature of his case must determine. In my own journey I met with many annoyances, which might have been readily enough avoided by a better arrangement, and these were well fitted to draw heavily on the strength which an invalid possesses, and sometimes must task it too severely. It is of course productive of mischief in many chronic enlargements of the viscera and subacute inflammations, in which motion aggravates the slow irritation,—but the rough journey, and the bracing air, are amongst the most powerful restoratives to the numerous class of dyspeptics and nervous patients who flock to the springs. In these persons the improvement is usually very manifest, and takes place as soon as the mountains are reached. The patients who belong to these classes are chiefly from the cities in the Northern and Middle States, or the low alluvial plains of the South; and in both of them much of the beneficial effects of the journey to the springs evidently arises from the total change in their habits of life, and the decided impression made upon their system by air and exercise totally different from their own. The peculiarities of their situation give the Virginia springs many advantages in the treatment of these affections, for there is no way of avoiding the mountain journeys, situated as the most of them are in the very heart of the mountain; you cross them, no matter in what way you may attempt to reach the springs; but if you come from the west to the White Sulphur, you avoid the highest mountain ranges, which lie between it and the Warm Springs, or between the Warm Springs and Charlottesville; still they are very fairly distributed.

The road which I selected was that by Charlottesville, following the great southern mail route through Baltimore and Washington to the junction of the Louisa rail-road, which branches off in a westerly direction towards Charlottesville, terminating about twenty miles to the east of this town; these twenty miles must, of course, be passed in stage-coaches, which were overcrowded on the day that it was my misfortune to enter, and as slow and tiresome as possible, so that the whole day was consumed before reaching Charlottesville; and instead of sleeping at Staunton, which is forty miles distant, the passengers who continued in the mail stages, travelled during the whole night; if any of them were so unfortunate as to be invalids, the toilsome journey in crowded coaches over the rough roads near the Blue Ridge must have been severe treatment. At Staunton, the two great roads from the east and north unite, and there remains but one day's journey to the Warm Springs.

An invalid should either not attempt to make the journey by the stage-coach, or he should at least reserve to himself the liberty of leaving it whenever he pleases; I mean, of course, a true invalid; many persons, who are either imaginary invalids, or labour under diseases which impair the strength but little, may, without fear, continue their journey. The climate changes as the mountains become nearer, and is sometimes quite cold in the mornings and evenings, even at midsummer; there is rarely any other inconvenience felt from the climate than a revival of the rheumatic pains, or occasionally an attack of diarrhœa,—these are in general very readily checked by a dose of laudanum, or some simple carminative.

The springs are usually visited in succession. The Warm Springs are first reached by the traveller from the east; the Hot Springs, at the distance of five miles; and thirty-five miles further to the west we find the White Sulphur; twenty-one miles further is the Salt Sulphur; and seventeen miles to the southwest is the Red Sulphur, which is the most distant of the springs. The Blue Sulphur and the Sweet Springs are not on the route from the Eastern cities to the Red Sulphur. I shall speak of these springs as I pass through them on my return.

